# The EPIYA-ABCC motif pattern in *CagA* of *Helicobacter pylori* is associated with peptic ulcer and gastric cancer in Mexican population

**DOI:** 10.1186/s12876-014-0223-9

**Published:** 2014-12-24

**Authors:** Fredy Omar Beltrán-Anaya, Tomás Manuel Poblete, Adolfo Román-Román, Salomón Reyes, José de Sampedro, Oscar Peralta-Zaragoza, Miguel Ángel Rodríguez, Oscar del Moral-Hernández, Berenice Illades-Aguiar, Gloria Fernández-Tilapa

**Affiliations:** Clinical Research Laboratory, Academic Unit of Chemical-Biological Sciences, Autonomous University of Guerrero, Chilpancingo, Guerrero C.P. 39090 Mexico; State Institute of Oncology “Dr. Arturo Beltrán Ortega”, Acapulco, Guerrero C. P. 39570 Mexico; General Hospital “Dr. Raymundo Abarca Alarcón”, Chilpancingo, Guerrero C.P. 39090 Mexico; Department of Chronic Infections and Cancer, Infectious Diseases Research Center, National Institute of Public Health, Av. Universidad No. 655, Cerrada los Pinos y Caminera, Colonia Santa María Ahuacatitlan, Cuernavaca, Morelos C.P. 62100 Mexico; Laboratory of Molecular Biomedicine, Academic Unit of Chemical-Biological Sciences, Autonomous University of Guerrero, Chilpancingo, Guerrero C.P. 39090 Mexico

**Keywords:** *cagA* gene 3′ region, CRPIA, EPIYA, CagA, *H. pylori*

## Abstract

**Background:**

*Helicobacter pylori* chronic infection is associated with chronic gastritis, peptic ulcer, and gastric cancer. Cytotoxin-associated gene A (*cagA*)-positive *H. pylori* strains increase the risk of gastric pathology. The carcinogenic potential of CagA is linked to its polymorphic EPIYA motif variants. The goals of this study were to investigate the frequency of *cagA*-positive *Helicobacter pylori* in Mexican patients with gastric pathologies and to assess the association of *cagA* EPIYA motif patterns with peptic ulcer and gastric cancer.

**Methods:**

A total of 499 patients were studied; of these, 402 had chronic gastritis, 77 had peptic ulcer, and 20 had gastric cancer. *H. pylori* DNA, *cagA*, and the EPIYA motifs were detected in total DNA from gastric biopsies by PCR. The type and number of EPIYA segments were determined by the electrophoretic patterns. To confirm the PCR results, 20 amplicons of the *cagA* 3′ variable region were sequenced, and analyzed *in silico*, and the amino acid sequence was predicted with MEGA software, version 5. The odds ratio (OR) was calculated to determine the associations between the EPIYA motif type and gastric pathology and between the number of EPIYA-C segments and peptic ulcers and gastric cancer.

**Results:**

*H. pylori* DNA was found in 287 (57.5%) of the 499 patients, and 214 (74%) of these patients were *cagA*-positive. The frequency of *cagA*-positive *H. pylori* was 74.6% (164/220) in chronic gastritis patients, 73.6% (39/53) in peptic ulcer patients, and 78.6% (11/14) in gastric cancer patients. The EPIYA-ABC pattern was more frequently observed in chronic gastritis patients (79.3%, 130/164), while the EPIYA-ABCC sequence was more frequently observed in peptic ulcer (64.1%, 25/39) and gastric cancer patients (54.5%, 6/11). However, the risks of peptic ulcer (OR = 7.0, 95% CI = 3.3–15.1; p < 0.001) and gastric cancer (OR = 5.9, 95% CI = 1.5–22.1) were significantly increased in individuals who harbored the EPIYA-ABCC *cagA* gene pattern.

**Conclusions:**

*cagA*-positive *H. pylori* is highly prevalent in southern Mexico, and all CagA variants were of the western type. The *cagA* alleles that code for EPIYA-ABCC motif patterns are associated with peptic ulcers and gastric cancer.

## Background

Chronic *Helicobacter pylori* infection is etiologically related to chronic gastritis, gastric ulcers, and gastric cancer [1-4]. Cytotoxin-associated gene A (CagA)-producing strains seem to induce gastrointestinal disease more frequently than non-producing strains [5,6]. While the presence of CagA does not explain the variability in the clinical results, this oncoprotein is associated with severe gastroduodenal pathology [7-15]. *CagA*-positive strains are known to induce more intense gastric mucosal inflammation compared to *cagA*-negative strains. This pro-inflammatory potential of *cagA*-positive *H. pylori* could explain its association with severe atrophic gastritis and gastric adenocarcinoma [16,17]*.* The CagA oncoprotein is released within epithelial cells via a type IV secretion system [18,19]. Upon translocation, CagA localizes to the internal surface of the plasma membrane, where it is phosphorylated on C-terminal variable region tyrosine residues by multiple host Src tyrosine kinase family member proteins [20-22]. The phosphorylation motifs are defined by the Glu-Pro-Ile-Tyr-Ala (EPIYA) sequence and are classified as EPIYA-A, B, C, or D according to the amino acids that flank these motifs. Western CagA strains have the A and B segments and 1 or more C segments. CagA strains from Eastern Asia have the A, B, and D segments. This explains the size variability of CagA proteins (range, 120–145 kDa) [3,9,23]. The main phosphorylation target in CagA is the tyrosine in the EPIYA-C and EPIYA-D motifs. The phosphorylation level is proportional to the number of EPIYA-C motifs, and thus, increased motif numbers increase the pro-inflammatory and carcinogenic potential of the protein. Phosphorylated CagA forms complexes with the SHP-2 phosphatase, resulting in abnormal signaling. This leads to subsequent cellular alterations that increase the risk of cells altered by pre-cancerous genetic changes [3,23-28]. In epithelial cells, SHP-2 binds more tightly to EPIYA-D than to EPIYA-C. However, CagA proteins with EPIYA-ABCCC have the same carcinogenic potential as those with EPIYA-D [25]. Western CagA-producing *H. pylori* strains with EPIYA-C sequences are more virulent and carcinogenic than CagA-producing strains with EPIYA-A and B motifs [15,29,30].

The prevalence of *cagA*-positive *H. pylori* is 90–95% in Asian countries and 50–60% in western countries [3,23]. *CagA* genotype distribution varies among regions and ethnic groups. For example, the Amerindian (AM) *cagA* allelic variants, which are found in the inhabitants of the Peruvian Shimaa village, encode CagA isoforms that contain altered or degenerate EPIYA-B motifs, specifically ESIYT in AM-I and GSIYD in AM-II. Additionally, the AM CagA contains attenuated conserved repeats that are responsible for phosphorylation-independent activity (CRPIA). The AM strains have attenuated proliferation and induce low-grade inflammation, resulting in low virulence and a decreased risk of severe pathology [26,31].

*CagA* is one of the most studied genes worldwide. In Mexico, the seroprevalence of *cagA*-positive *H. pylori* varies between 40% and 90% in patients with gastric pathology from different zones throughout the country [8,32-36]. In patients from Mexico City who presented gastroduodenal pathology, the EPIYA segments of *cagA*-positive strain were of the western type [37]. In another study conducted in children with abdominal pain and adults with duodenal ulcers, gastric ulcers, or non-ulcerous dyspepsia, the identified EPIYA patterns were ACC, ABC, ABCC, ABCCC, and ABABC [37]. The following sequences were identified in gastric cancer and chronic gastritis cases: ABC, ABCC, ABABC, AABCC, and ABCCC [38]. However, to date, no studies have been conducted to explore the association between the type and number of EPIYA segments and severe gastric pathologies in southern Mexico. The analysis of the association between the EPIYA-C motif number and peptic ulcers and gastric cancer, will help to clarify the relationship between CagA variants and gastric disease severity in *H. pylori*-infected Mexican patients. The goal of this study was to investigate the prevalence of *cagA*-positive *H. pylori* and the EPIYA motif types in the gastric mucosa of patients with chronic gastritis, peptic ulcers, and gastric cancer to determine whether the EPIYA-C motif number is associated with ulcers and gastric cancer.

In this study, we found a high prevalence of western-type *cagA*-positive *H. pylori* infection, with a predominant EPIYA-ABCC pattern in Mexican patients with peptic ulcers and gastric cancer. Interestingly, the presence of a CagA protein with 2 or more EPIYA-C motifs was associated with severe gastric pathology.

## Methods

### Patients

A total of 499 patients were studied. The study subjects were sequentially selected from patients who suffered from dyspepsia symptoms and had been subjected to upper gastrointestinal tract endoscopy at the Chilpancingo’s General Hospital “Dr. Raymundo Abarca Alarcón” or at the State Institute of Oncology in Acapulco, Guerrero, Mexico. The subjects were recruited between April 18, 2007 and April 19, 2013. Patients in this study had not received treatment with anti-microbial agents, proton pump inhibitors, or gastric pH-neutralizing agents for a month before the endoscopic treatment. Patients who received immunosuppressive or non-steroid anti-inflammatory treatment were excluded from the study. Either the patients or their parents signed an informed consent letter. This project was approved by the Bioethics Committee of the Autonomous University of Guerrero and the participating hospitals.

### Biopsy collection

Endoscopies were conducted after an overnight fast with a video processor and a video gastroscope (Fujinon, Wayne, NJ, USA). Two biopsies from the gastric antrum or body, the ulcer edge, or the tumor were collected. One biopsy was immediately fixed in 10% formalin for histological analysis, while the other was placed in a buffered solution (10 mM Tris, pH 8.0, 20 mM EDTA, pH 8.0, 0.5% SDS) for the molecular diagnosis of *H. pylori*. The latter biopsies were stored at −20°C until processing.

### Histology

The formalin-fixed biopsies were embedded in paraffin, and 4-μm sections were stained with hematoxylin-eosin for histological analysis. Histopathological findings were used to determine each patient’s diagnosis. Gastritis was classified according to the updated Sydney system.

### *H. pylori* detection

Total DNA was extracted from gastric biopsies according to the phenol-chloroform-iso-amyl alcohol technique after proteinase K digestion [39]. The specific presence of the *H. pylori* 16S rRNA gene was assessed according to the methods previously described by Román-Román *et al*. [40]. For all reactions, DNA samples from the *cagA*-positive ATCC43504 and J99 *H. pylori* strains were used as positive controls. For negative controls, DNA was substituted with sterile deionized water. All reactions were performed in a Mastercycler Ep gradient thermocycler (Eppendorf, Hamburg, Germany).

### *CagA* gene amplification

*H. pylori* 16S rRNA gene-positive samples were subjected to PCR to detect the *cagA* gene using the primers described previously by Figura *et al.,* [9]. These oligonucleotides amplified a 298-bp fragment within the constant region [9]. To amplify a 550- to 850-bp region within the 3′ variable region of the *cagA* gene the primers cag2 and cag4 described previously by Argent *et al.,* were using [41,42], Table 1. The reaction mix consisted of 1.7 mM MgCl2, 0.2 mM dNTPs (Invitrogen, Carlsbad, CA, USA), 5 pmol of each oligonucleotide, 1 U of Platinum® Taq DNA polymerase (Invitrogen Carlsbad, CA, USA), and 300 ng of total DNA in a total volume of 25 μl. The following amplification conditions were used: 1 cycle at 94°C for 5 min; 30 cycles at 94°C for 40 s, 56°C for 30 s, and 72°C for 50 s; and a final extension cycle at 72°C for 10 min. The PCR products were subjected to electrophoresis on a 1.5% agarose gel, followed by ethidium bromide staining and analysis under an ultraviolet (UV) light. Samples were considered CagA-positive when at least 1 of the 2 bands was observed.

**Table 1:** **PCR primers used in this study**

**Primer name and reference**	**Primer sequence (5′to 3′)**	**Motif amplied**	**Size (bp)**
cagAF D008 [9]	ACAATGCTAAATTAGACAACTTGAGCGA	Constant region of the *cag*A gene	298
cagAR R008 [9]	TTAGAATAATCAACAAACATCACGCCAT		
cag2F [30,41]	GGAACCCTAGTCGGTAATG	*cagA* 3′ variable region	550 to 850
cag4 [30,41]	ATCTTTGAGCTTGTCTATCG		
*cagA*28F [41]	TTCTCAAAGGAGCAATTGGC	Forward for all EPIYA motifs	
*cagA-*P1C [41,42]	GTCCTGCTTTCTTTTTATTAACTTKAGC	*EPIYA-A*	*264*
*cagA-*P2TA [41,42]	TTTAGCAACTTGAGTATAAATGGG	*EPIYA-B*	*306*
*cagA*West [42]	TTTCAAAGGGAAAGGTCCGCC	*EPIYA-C*	*501*
*cagA*East [42]	AGAGGGAAGCCTGCTTGATT	*EPIYA-D*	*495*

### Amplification of the *cagA* gene 3′ variable region and EPIYA motif prediction

Each *cagA*-positive sample was subjected to 4 PCR reactions to identify the EPIYA motifs. The sense oligonucleotide primer cag28F was used in all 4 reactions, while the antisense oligonucleotide primers cagA-P1C, cagAP2TA [41], CagAWest, and CagAEast [42] were used in separate reactions to amplify the EPIYA-A (~264 bp), B (~306 bp), C (~501 bp), and D (495 bp) motifs, respectively, Table 1. All PCR samples were prepared with 0.2 mM dNTPs (Invitrogen Carlsbad, CA, USA), 1.5 mM MgCl_2_, 10 pmol of each oligonucleotide, 1 U of Platinum® Taq DNA Polymerase (Invitrogen Carlsbad, CA, USA), and 300 ng of total gastric biopsy DNA in a final volume of 25 μl. The following amplification conditions were used: 1 cycle at 94°C for 5 min; 35 cycles at 94°C for 1 min, 58°C for 30 s, and 72°C for 1 min; and a final extension cycle at 72°C for 10 min. The PCR products were separated by electrophoresis on a 1.5% agarose gel, followed by ethidium bromide staining and UV light analysis.

### Sequencing and bioinformatics analysis of the *cagA* gene 3′ variable region

A subset of 20 samples was randomly selected for sequencing to confirm the PCR results. Cag28F and cag4 primers were used to amplify the variable region and generate ~650 to ~850-bp amplicons. The PCR reaction was conducted in a 50-μl volume with 15 pmol of each primer, 0.3 mM dNTPs, 2 mM MgCl_2_, and 1 U of Platinum® Taq DNA Polymerase (Invitrogen Carlsbad, CA, USA) per reaction. The amplification conditions were as follows: 1 cycle at 94°C for 5 min; 30 cycles at 94°C for 40 s, 55.5°C for 30 s, and 72°C for 50 s; and a final extension cycle at 72°C for 7 min. The PCR products were purified with the PureLink® PCR Purification Kit (Invitrogen Carlsbad, CA, USA) according to the manufacturer’s instructions. The purified products were sequenced with the BigDye terminator v1.1 sequencing kit (Applied Biosystems, Foster City, CA, USA) and analyzed with an ABI PRISM 310 Genetic Analyzer (Applied Biosystems). The nucleotide sequences were transformed into amino acid sequences with MEGA v5 software [43]. The ClustalW option within the MEGA software was used to generate a multiple amino acid sequence alignment. The partial CagA protein sequence from the *H. pylori* strain 43526 (GenBank: AF001357.1) was used as a reference.

### Statistical analysis

Kruskal-Wallis, ANOVA, χ^2^, and Fisher’s exact test analyses were used to determine significant differences. Associations between the presence of *H. pylori*, CagA, and the EPIYA-C motif number were determined in multinomial logistic regression models at a confidence interval of 95%. A p-value < 0.05 indicated statistical significance. All analyses were conducted with the Stata v11.1 software package (StataCorp, College Station, TX, USA).

## Results

### Population characteristics

Of the 499 studied patients, 402 (80.6%) were diagnosed with chronic gastritis, 77 (15.4%) with peptic ulcers, and 20 (4%) with gastric cancer. The age of patients ranged from 11 to 80 years old. The cancer patients were significantly older (p < 0.001) than those in the other groups, and the female gender was predominant in all 3 groups. Education years were significantly different among the groups (p < 0.001), Table 2.

**Table 2:** **Sociodemographic characteristics in Mexican patients with chronic gastritis, peptic ulcers, and gastric cancer**

	**Diagnosis**			
	**Chronic gastritis** **n = 402**	**Peptic ulcer** **n = 77**	**Gastric cancer** **n = 20**	***p*** **value**
**Age** (mean ± SD)	47.4 ± 16.7	52.8 ± 16.5	58.7 ± 16)	0.0009^†^
**Gender** n (%)				
Male	155 (38.6)	33 (42.9)	9 (45)	0.682^◊^
Female	247 (61.4)	44 (57.1)	11 (55)	
**Smoking habit** n (%)				
No	239 (59.5)	36 (41.8)	10 (50)	0.096^◊^
Current smoker or former smoker	163 (40.5)	41 (53.2)	10 (50)	
**Alcohol consumption** n (%)				
No	100 (24.9)	22 (28.6)	6 (30)	0.716^◊^
Consumes or consumed	302 (75.1)	55 (71.4)	14 (70)	
**Education** [median (ranges), years]	12 (6–17)	12 (6–17)	6 (0–7.5)	0.0001^▀^

†ANOVA test; ^▀^Kruskal-Wallis test; ◊ χ^2^ test.

### *CagA* status of *Helicobacter pylori* infections

**Figure 1:**
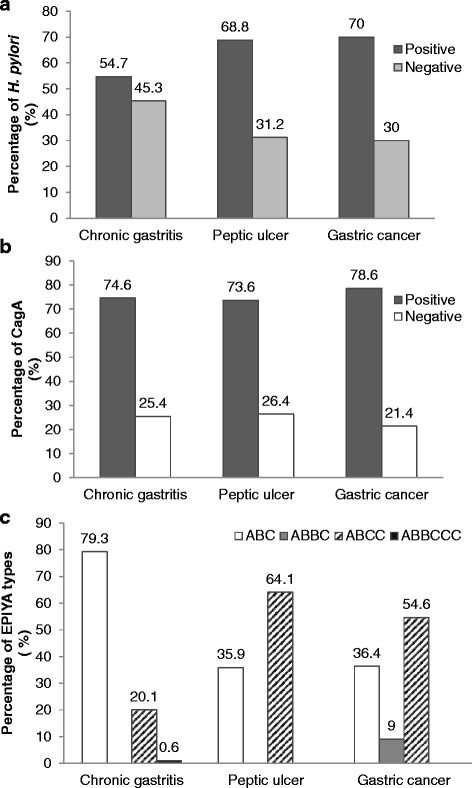
**Prevalence of** ***H. pylori, cagA*** **, and EPIYA patterns according to histopathological diagnoses. A**) Percentage of patients with *H. pylori* infection according to gastric disease. There were statistically significant differences in the prevalence of *H. pylori* among the study groups (p = 0.037, χ^2^ test). **B**) Percentage of *cagA* among patients with *H. pylori* infection. The prevalence of *cagA*-positive *H. pylori* was very similar among the study groups (p = 0.930, χ^2^ test). **C**) The prevalence of the different EPIYA patterns in the *cagA* gene is shown. The EPIYA-ABC and ABCC sequences were differentially distributed among patients with chronic gastritis, peptic ulcers, and gastric cancer (p = 0.000; Fisher’s exact test).

The presence of the *H. pylori* 16S rRNA gene was detected in the gastric mucosa samples from 287 (57.5%) patients. The difference in the infection frequencies according to the diagnosis was significant (p = 0.037), and a higher prevalence was observed in gastric cancer patients (70%), Figure 1A. The *H. pylori cagA* gene was found in 214 (74%) of the 287 infected patients, Figure 1B. However, no significant differences in the frequencies of *cagA*-positive *H. pylori* were found among the study groups (p = 0.930). The congruence between the two PCR assays for determining *cagA* status was 88% (Kappa correlation coefficient = 0.8857 p <0.001), data not shown

### EPIYA segments and EPIYA-C motif numbers

**Figure 2:**
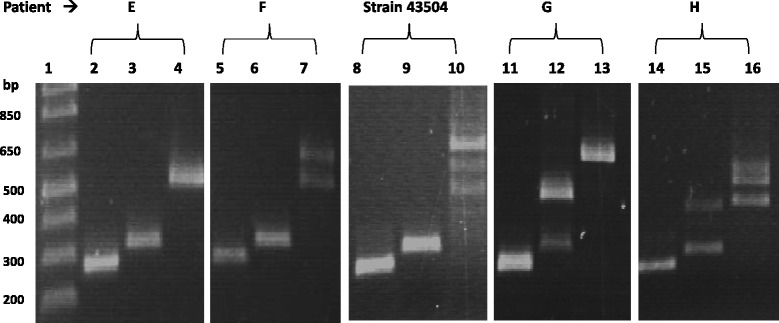
**Electrophoresis of representative samples with different CagA EPIYA patterns.** DNA from representative clinical samples from *cagA*-positive *H. pylori* patients (E, F, G, H) was amplified by EPIYA motif-specific PCR. The PCR products were analyzed on a 1.5% agarose gel. Column 1: 100 bp MW marker; E (columns 2–4) EPIYA-ABC; F (columns 5–7) EPIYA-ABCC; G (columns 11–13) EPIYA-ABBC; H (columns 14–16) EPIYA-ABBCCC. DNA from the *H. pylori* 43504 strain, which contains the EPIYA-ABCCC motif, was used as a positive control. Size of products of EPIYA motifs by PCR-specific: EPIYA A motif (~264 bp), EPIYA B (~306 bp ), second EPIYA B motif (500 bp), first EPIYA C (501 PB) second EPIYA C motif (~650 bp) and third EPIYA-C (>650 bp).

The PCR products amplified from *cagA*-positive samples showed four electrophoretic patterns that corresponded to the following combinations of EPIYA motifs: ABC, ABCC, ABBC, and ABBCCC. The EPIYA-D motif was not detected, Figure 2.

The EPIYA-ABC segment was detected in 148 (69.2%) patients, while the ABCC motif was detected in 64 (29.9%) of the 214 *H. pylori cagA*-positive subjects. The EPIYA ABBCCC motif was only detected in one patient with chronic gastritis, while the ABBC motif was only detected in one patient with gastric cancer, Figure 1C. The EPIYA-ABC pattern was found in 130 (79.3%) of the 164 *cagA*-positive *H. pylori* patients with chronic gastritis and was more frequent in this group than in ulcer and cancer. *Cag*A-positive *H. pylori* with two EPIYA-C motifs was more frequently detected in patients with ulcers and gastric cancer (64.1% and 54.6%, respectively), Table 3, Figure 1C. The results were confirmed by sequencing a ~650- to ~850-bp fragment within the 3′ variable region of the *cagA* gene in 20 randomly selected samples. The agreement between the results of PCR and sequencing was 100%.

**Table 3:** **Association of H. pylori, cagA and EPIYA-C motif number with chronic gastritis, peptic ulcer and gastric cancer**

	***H. pylori***			
**Diagnosis**	**Negative**	**Positive**	**OR**	**CI 95%**
G	182	220	1.0	-
PU	24	53	1.8^c^	1.0-3.0
GC	6	14	1.9	0.72-5.1
Total	**212**	**287**		
	**CagA**			
	**Negative**	**Positive**	**OR**	**CI 95%**
G	56	164	1.0	-
PU	14	39	0.9	0.5- 1.9
GC	3	11	1.2	0.3 – 4.6
**Total**	**73**	**214**		
	**EPIYA motif**			
	ABC	ABCC	OR	IC95%
G	130	33	1.0	-
PU	14	25	7.0^a^	3.3 – 15.1
GC	4	6	5.9^b^	1.5-22.1
**Total**	**148**	**64**		
	**Number of EPIYA-C**			
	1 C^*^	≥2 C^ϕ^	OR	IC95%
G	130	34	1.0	-
PU	14	25	6.8^a^	3.2-15.6
GC	5	6	4.5^c^	1.3-15.9
**Total**	**149**	**65**		

G; chronic gastritis, UP peptic ulcer, CG: gastric cancer. ^a^p < 0.001 ; ^b^p <0.01; ^c^p < 0.05. * The EPIYA-ABBC was added; ^ϕ^ the EPIYA-ABBCCC was added

Note: Only the most frequent EPIYA motifs were considered.

### Bioinformatic analysis of the CagA amino acid sequence

**Figure 3:**
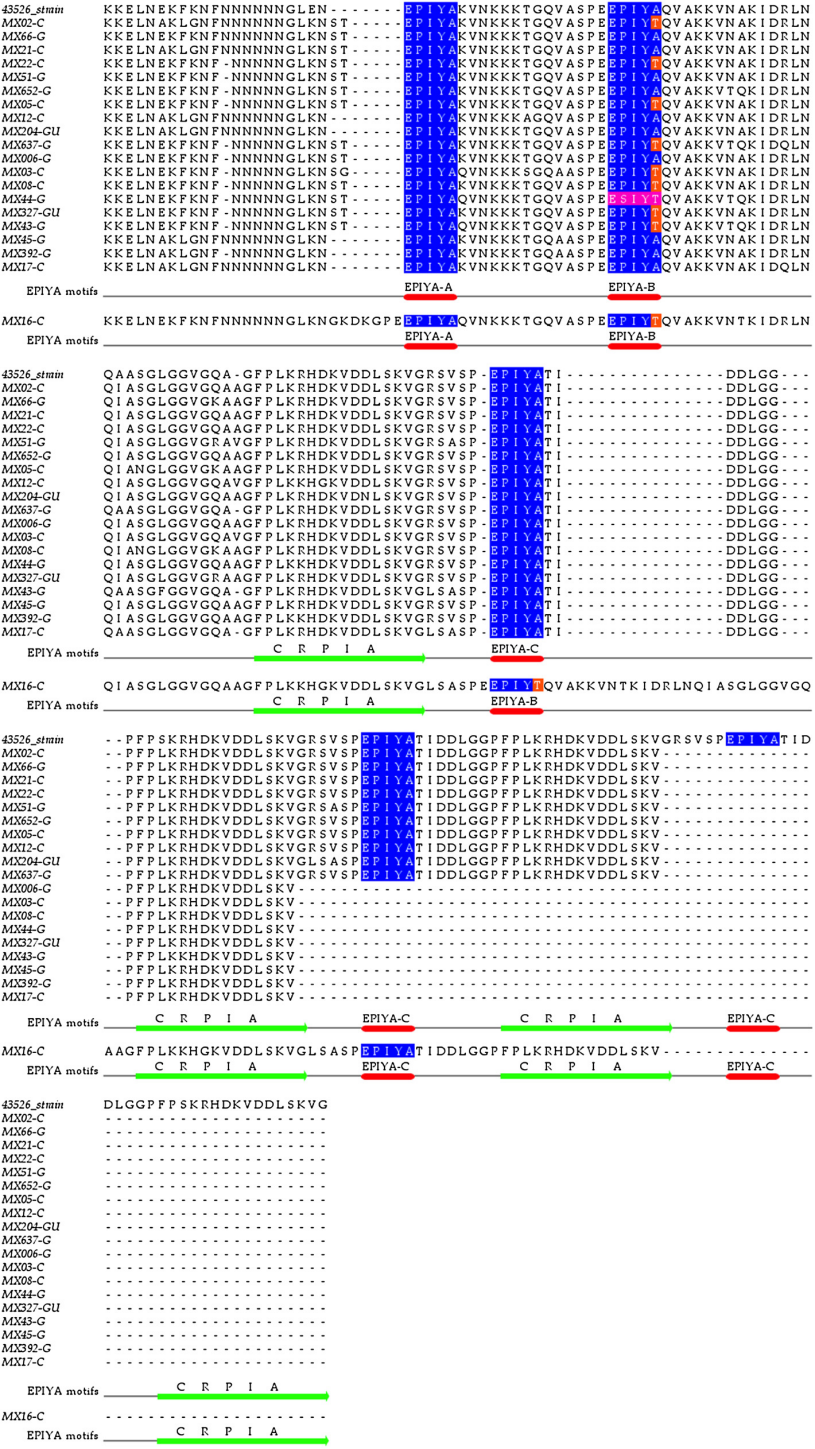
**Alignment of CagA sequences from patients with gastric disorders.** CagA amino acid sequences obtained from nine patients with chronic gastritis (G), two with peptic ulcer (GU) and nine with gastric cancer (C) are aligned with the CagA sequence from *H. pylori* reference strain 43526. The sample number is followed by the histological diagnosis. The EPIYA amino acids are shown in blue. The red lines highlight each EPIYA pattern, and the green lines underline the CRPIA motifs. Alanine-to-threonine changes (EPIYT) in EPIYA-B are shown in orange. The ESIYT sequence that corresponds to the proline-to-serine and alanine-to-serine changes in EPIYA-B is shown in pink. An AM-I strain was detected in one patient with chronic gastritis (MX44-G [GenBank: KF800906.1]. The sample MX16-C [GenBank: KF800911.1] from patient with gastric cancer contain a CRPIA motif in the N-terminus of its second EPIYA-B. This sequence is unique in that it contain an extra EPIYA-B motif and an extra CRPIA motif, making it difficult to align it with CagA from strain 43526. GenBank accession numbers of each strain in this figure:MX02A-C [KF800917.1], MX66-G [KF800903.1], MX21-C[KF800909.1], MX22-C[KF800908.1 ], MX51-G[KF800904.1], MX652-G[KF800898.1], MX05-C[ KF800915.1], MX12-C[KF800912.1], MX204-GU[KF800902.1], MX637-G[ KF800899.1], MX006-G[KF800914.1], MX03-C[ KF800916.1], MX08T-C[KF800913.1], MX44-G[KF800906.1], MX327-GU[KF800901.1], MX43-G[KF800907.1], MX45-G[KF800905.1], MX392-G[KF800900.1], MX17-C[KF800910.1], MX16-C[KF800911.1].

The *cagA* DNA sequences from the following amplicons were analyzed: 9 gastric cancer amplicons (MX02-C, MX21-C, MX22-C, MX05-C, MX12-C, MX03-C, MX08-C, MX17-C, MX16-C); 9 chronic gastritis amplicons (MX66-G, MX51-G, MX52-G, MX637-G, MX006-G, MX44-G, MX43-G, MX45-G, MX392-G) and two peptic ulcer amplicons (MX204-GU, MX327-GU). An *in silico* amino acid prediction was conducted to identify the EPIYA and CagA multimerization (CM, also known as CRPIA) motifs [26]. The following motifs and corresponding patterns were found: EPIYA-A with the EPIYA(K/Q)VNKKK (A/T/V/S)GQ pattern, EPIYA-B with the E(P/S)IY(A/T)(Q/K)VAKKV(N/T)(A/Q)KI pattern, and EPIYA-C with the EPIYATIDDLGGP pattern, Figure 3. Two EPIYA-B motif variants were found; one chronic gastritis sample (MX44-G) had an ESIYT sequence, while 10 (50%) of the 20 sequences contained the EPIYT pattern (MX02-C, MX22-C, MX05-C, MX637-G, MX03-C, MX08-C, MX327-GU, MX43-G, MX16-C). The following changes were found among the 16 amino acid residues that comprise the CRPIA motif: FPLK(R/K)H(D/G)KVD(D/N)LSKVG for the first CRPIA motif in the N-terminus of EPIYA-C, FPLK(R/K)H(D/G)KVDDLSKVG for the second CRPIA motif, and FPLKRHDKVDDLSKV for the last CRPIA motif in the C-terminus. A CRPIA motif was identified in the N-terminus of one of the two EPIYA-B motifs in the CagA-containing MX16-C gastric cancer sample. Within this sequence, the amino acids GKDKGPE were found in the N-terminus of the EPIYA-A motif (Figure 3). All CRPIA motifs were of the western type, Figure 3. Nucleotide and predict protein sequences of all strains were deposited in GenBank, accession numbers [GenBank:KF800898.1- GenBank:KF800917.1]

.

### Association between *H. pylori* infection, *cagA*-positive strains, peptic ulcers, and gastric cancer

*H. pylori* infection was associated with peptic ulcers (OR = 1.8; 95% CI = 1.0–3.0) but not with gastric cancer (OR = 1.9; 95% CI = 0.72–5.1). On the other hand, infection with *cagA*-positive strains was not associated with either ulcers or gastric cancer, Table 3.

### Association between the EPIYA-C motifs number with peptic ulcers, and gastric cancer

The presence of the EPIYA-ABCC segment was associated with peptic ulcers (OR = 7.0; 95% CI = 3.3–15.1; p < 0.001) and gastric cancer (OR = 5.9; 95% CI = 1.5–22.1; p = 0.008). The increase in the number of EPIYA-C repeats was also associated with peptic ulcers (OR = 6.8; 95% CI = 3.2–15.6; p < 0.001), as well as cancer (OR = 4.5; 95% CI = 1.3-15.9; p = 0.017), Table 3.

## Discussion

Infection with a *cagA*-positive *H. pylori* strain is recognized as the most important risk factor for gastric cancer and is also associated with atrophic gastritis and duodenal ulcers [29,44]. Nonetheless, the majority of infected patients do not develop serious diseases.

In the present study, we found that 57.5% of patients with gastric pathologies were *H. pylori*-positive, and 74% of the infecting strains harbored the *cagA* gene. The global prevalence of *cagA*-positive *H. pylori*, which ranges from 43% to 90%, is in accordance with the previously reported seroprevalence in a Mexican population with gastric pathologies [8,32-36]. However, serology might overestimate the frequency of *H. pylori* and *cagA*-positive strains as it is unable to differentiate between current and past infections. The discrepancies in the prevalence of *H. pylori* can be explained by differences in the diagnostic method used, age of patients, geographic area and the environmental health conditions in which people live. Another possible explanation is that the rate of infection is decreasing [36]. In our study, the prevalence of *cagA*-positive *H. pylori* in patients with chronic gastritis was higher (74.6%) than the rate reported in 2009 by Paniagua *et al.* (52.4%) via multiplex PCR [45]. In gastric cancer patients, the prevalence of *cag*A was higher (78.6%) than the seroprevalence reported in 2008 by Carmolinga *et al*. (66.2%) [8]. In this work, the frequency of *H. pylori cagA*-positive that we found was similar to antibodies prevalence in Mexican subjects and, unlike to other studies, the strengths of our study are in the sample size and the high sensitivity and specificity of the methods used to detect *H. pylori* and *cagA*.

Some authors have found an association between CagA and the severity of gastric pathologies [7,11-14]. It has been proposed that this relationship might be explained by the number of EPIYA-C motifs in the protein as these motifs influence the degree of virulence and oncogenic potential of *cagA*-positive *H. pylori* [7,46]. It is likely that determining the EPIYA motifs in CagA, rather than detecting *cagA* per se, would be a better marker for assessing the risk of serious gastric pathology [41,47]. In our study, 100% of the EPIYA motifs identified in CagA were of the western type, and their distributions among the pathologies were significantly different (p ≤ 0.001). In 69.1% of the cases, the *cag*A gene contained an EPIYA-C motif in the typical ABC sequence, and this was more frequent in patients with chronic gastritis (79.3%). This result was similar to that reported by Batista *et al*. for Brazilian populations (70.6% in total of cases and 79.4% in patients with gastritis), [48] but higher than that found in Colombian patients by Quiroga *et al.* (49% in total of cases and 59.6% in patients with gastriris), [29] and by Acosta *et al.* (62.3% in total of cases and 52.6%, in gastritis) [49]. Interestingly, Rizzato *et al*. [38] detected the EPIYA-ABC pattern in 82% of Venezuelan and Mexican patients with chronic gastritis and gastric cancer, without finding frequency differences between the groups. Reyes-León *et al.* [37] found that the ABC sequence was more frequent (50%) in children from Mexico City with chronic abdominal pain. These findings emphasize the differences in the geographic distribution of *H. pylori* strains, and these differences might be related to the uneven prevalence of gastric cancer in the inhabitants of different Mexican regions.

The frequency of cases that harbored *cagA*-positive *H. pylori* with two EPIYA-C motifs was higher in gastric cancer patients (64%) and thus higher than the frequencies reported by Acosta (27.7%) and Quiroga (35.3%) in Colombia and by Batista in Brazil (34.6%) [29,48,49]. We found that the *cagA* allele that encoded two EPIYA-C segments was also predominant in patients with peptic ulcers (54.5%). This frequency is higher than that reported by Torres in Cuban patients (15.7%) [50]. Unexpectedly, the only motif with three EPIYA-C repeats (ABBCCC) was found in a patient with chronic gastritis. Reportedly, an increase in the number of phosphorylation sites in the C-terminus of CagA is associated with the carcinogenic potential of *H. pylori* [15,37,49]. Thus, it is likely that those patients with chronic gastritis infected with a *H. pylori* strain with *cagA* gene that encodes two or more EPIYA-C motifs (21%) are at higher risk of developing more serious diseases.

The amino acid sequences obtained during a bioinformatics analysis revealed that the alanine-to-threonine substitution in the EPIYA-B (EPIYT) motif occurred frequently (10 out of 20 sequences) in the studied groups. These findings are in accordance with those reported by Rizzato *et al*. in 2012 for Mexican and Venezuelan subjects with chronic gastritis and gastric cancer (50% of the B motifs harbored the EPIYT variant, with no significant differences between the groups) [38]. ABCC isolates bearing this modification have also been reported to cause decreased levels of cellular elongation and IL-8 secretion compared to those that bear the normal ABCC pattern [37]. It is possible that, in a Mexican population, the frequency of CagA isoforms with the EPIYT amino acid sequence in EPIYA-B is associated with the prevalence of gastroduodenal diseases. However, the existing epidemiological and experimental studies are insufficient to further support this hypothesis.

The ESIYT modification in EPIYA-B was identified in one chronic gastritis sample. This sequence belonged to the AM-I CagA variant, which has been associated with low *H. pylori* virulence in comparison to the western or Asian strains [26]. However, the AM-I and II CagA variants, such as those found in indigenous Mexican groups with Amerindian ancestry, show degeneration or elimination in their CRPIA motifs [26,31,51]. Interestingly, the CagA variants with the ESIYT sequence found in the present study contained western-type CRPIA and therefore differed from the Amerindian variants [31]. A CRPIA sequence in the N-terminus of the EPIYA-B motif was also detected in a gastric cancer sample. This finding agrees with those reported by Sicinschi *et al.,* Sgouras *et al*. and Acosta *et al*., who noted that in some CagA variants, the CRPIA segment can be found in the N-termini of the EPIYA-A and B motifs [15,49,52]. The localization of the CRPIA motif within EPIYA-B might result from recombination between *H. pylori* strains with different *cagA* allelotypes or from the insertion of DNA sequences that contribute to *H. pylori* diversification [53,54]. The CRPIA sequences stabilize the CagA protein, influence its half-life and are associated with oncoprotein activity in epithelial cells [15]. Thus, our results highlight the need to evaluate the functional importance of the EPIYT and ESIYT variants in EPIYA-B. Furthermore, it is necessary to assess the effects that the observed sequence variants and the localization of the CRPIA motifs in the ABCC pattern exert on CagA activity. It is likely that the prevalence of some of these variants could explain why the gastric cancer incidence rates of male and female Mexican patients (9.4 and 6.7/100,000, respectively) are similar to those reported in Southeastern Asian countries (10.2 and 4.7/100,000 in men and women, respectively) [55], despite the differences in the *cagA*-positive *H. pylori* prevalence (90–95% in Japan, Korea, and China; 50–60% in Mexico).

The association of *cagA* polymorphisms with severe gastric pathologies [7,29,48-50,52] or with pre-cancerous lesions is controversial [10,15,30,32], and only a few studies have been conducted in Hispanic populations with gastric ulcers [50]. This is the first study to investigate the prevalence of *cagA* variants in southern Mexico. Our results show that the presence of two or more EPIYA-C repeats within the *cagA* gene represents a higher risk of peptic ulcers and gastric cancer. It is likely that this increase in the number of EPIYA-C repeats plays an important role in the development of such diseases in individuals from this particular geographic region. A total of 51.5% of the samples with two EPIYA-C repeats came from patients with chronic gastritis. It is likely that some of these individuals have a higher risk of cancer development [29] given that the increase in the number of EPIYA-C motifs increases the CagA phosphorylation status and its interactions with cellular proteins that induce epithelial cell elongation, cell turnover, and pro-inflammatory cytokine production, thus facilitating the development of gastric cancer [29,41]. These findings might also be related to other clinical results.

The virulence factors of *H. pylori* are important risk determinants but are not sufficient to induce the full development of severe gastroduodenal disease. The host’s genetic and sociocultural factors also contribute to the risk of pre-cancerous lesions and gastric cancer [56,57].

## Conclusions

In conclusion, the present study shows that *cagA*-positive *H. pylori* infection is highly prevalent in patients from southern Mexico with chronic gastritis, peptic ulcers, and gastric cancer. All CagA isoforms were of the western type. The *cagA* allele that encodes the EPIYA-ABC pattern was most frequently observed in chronic gastritis samples, while the EPIYA-ABCC isoform predominated in peptic ulcer and gastric cancer samples. *CagA* variants that encode two or more EPIYA-C motifs are associated with peptic ulcer and gastric cancer. Likely, either the EPIYA-ABCC sequence or patterns with two or more EPIYA-C motifs are a risk marker for severe gastric pathologies.
